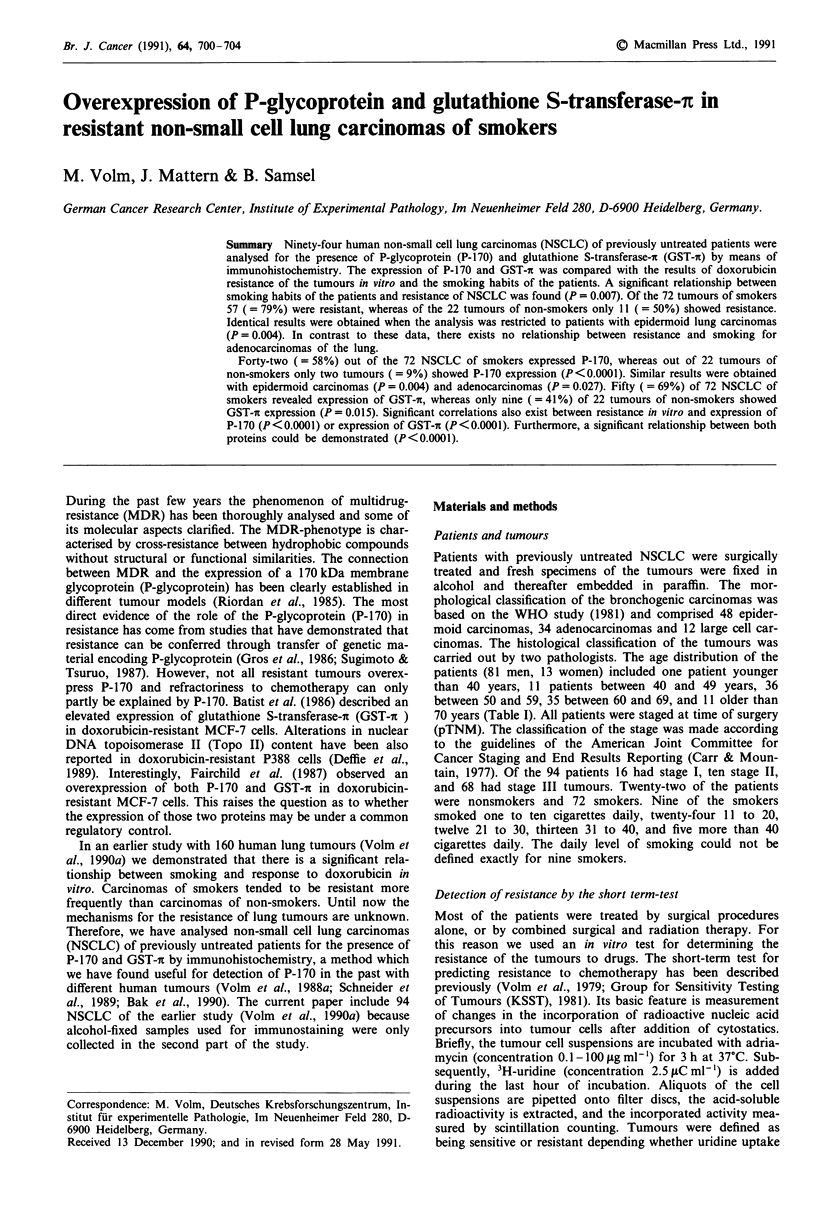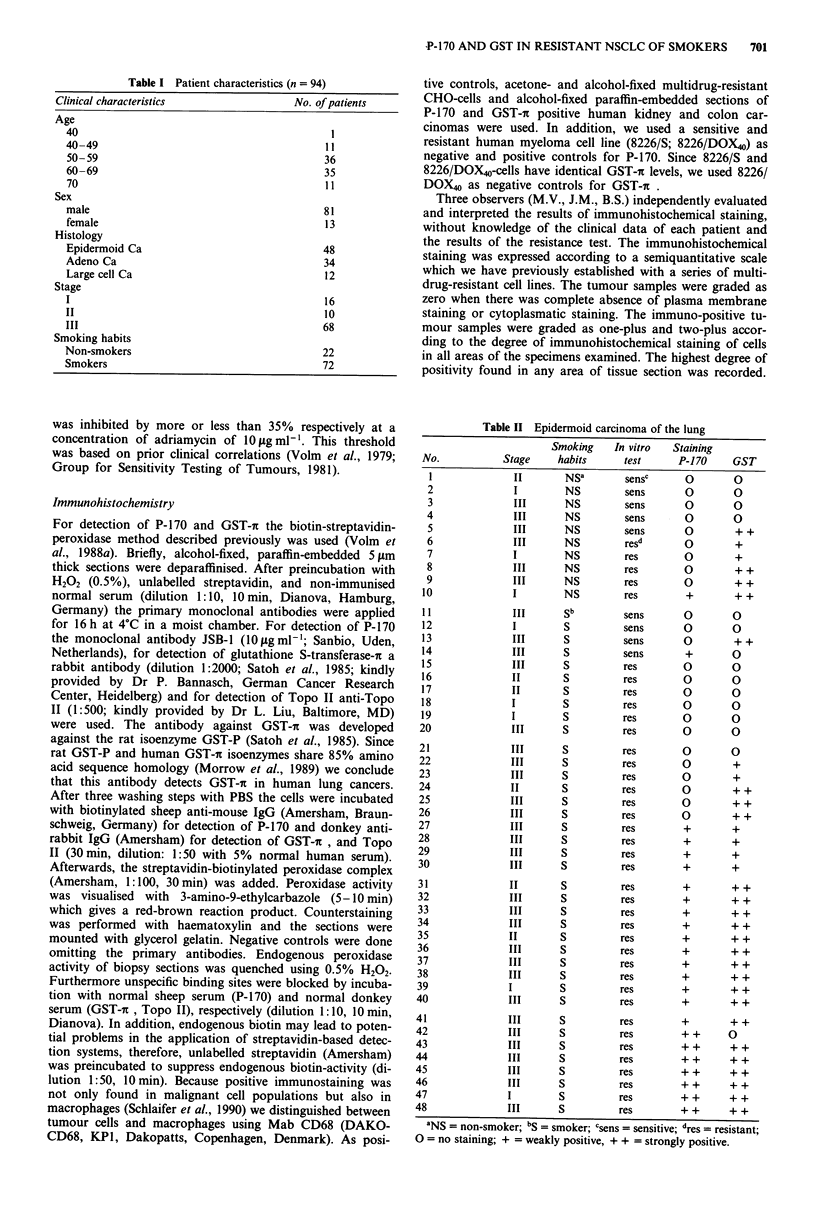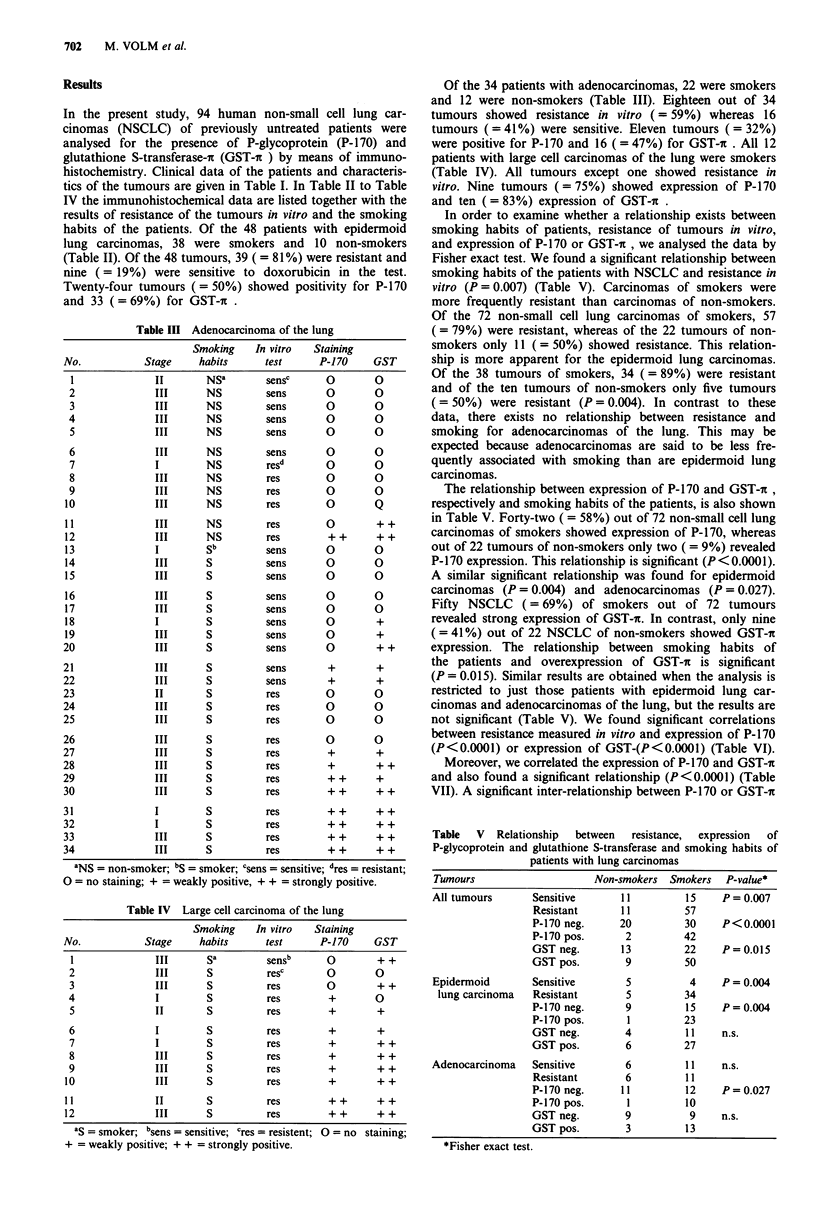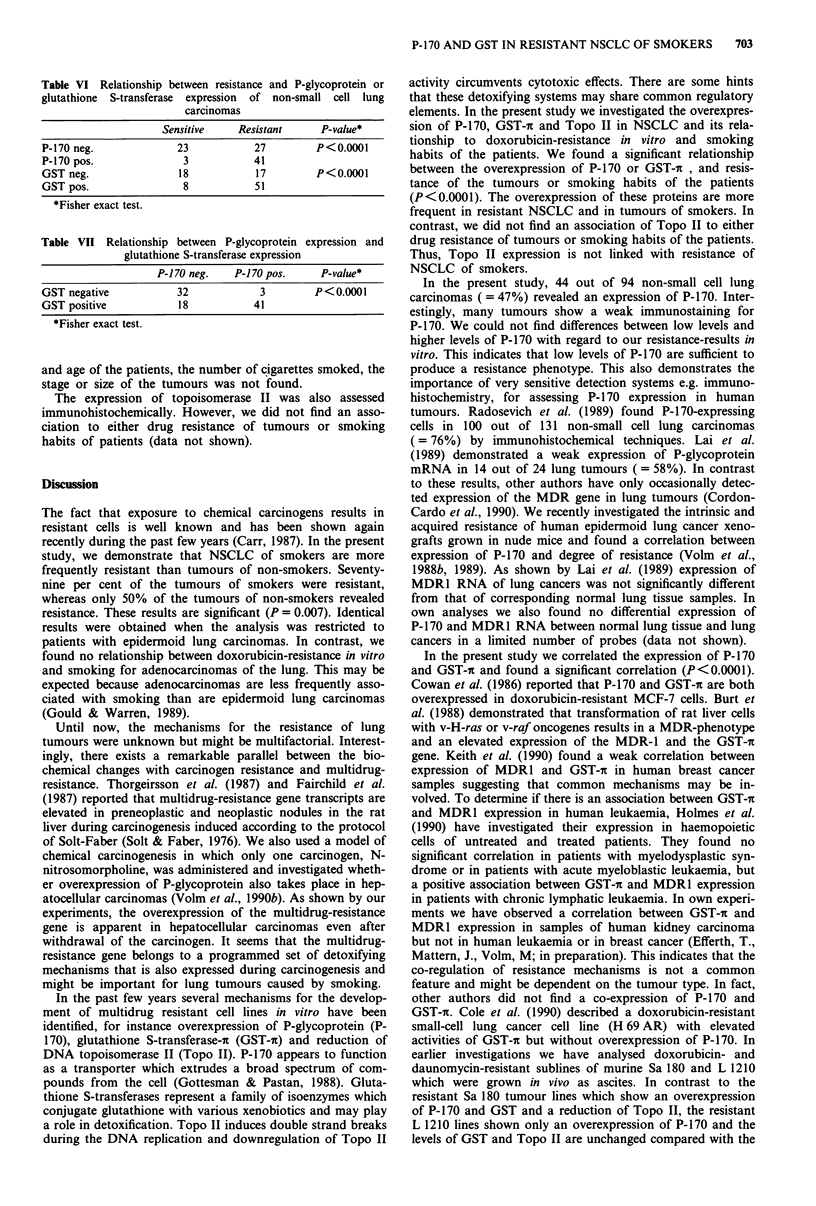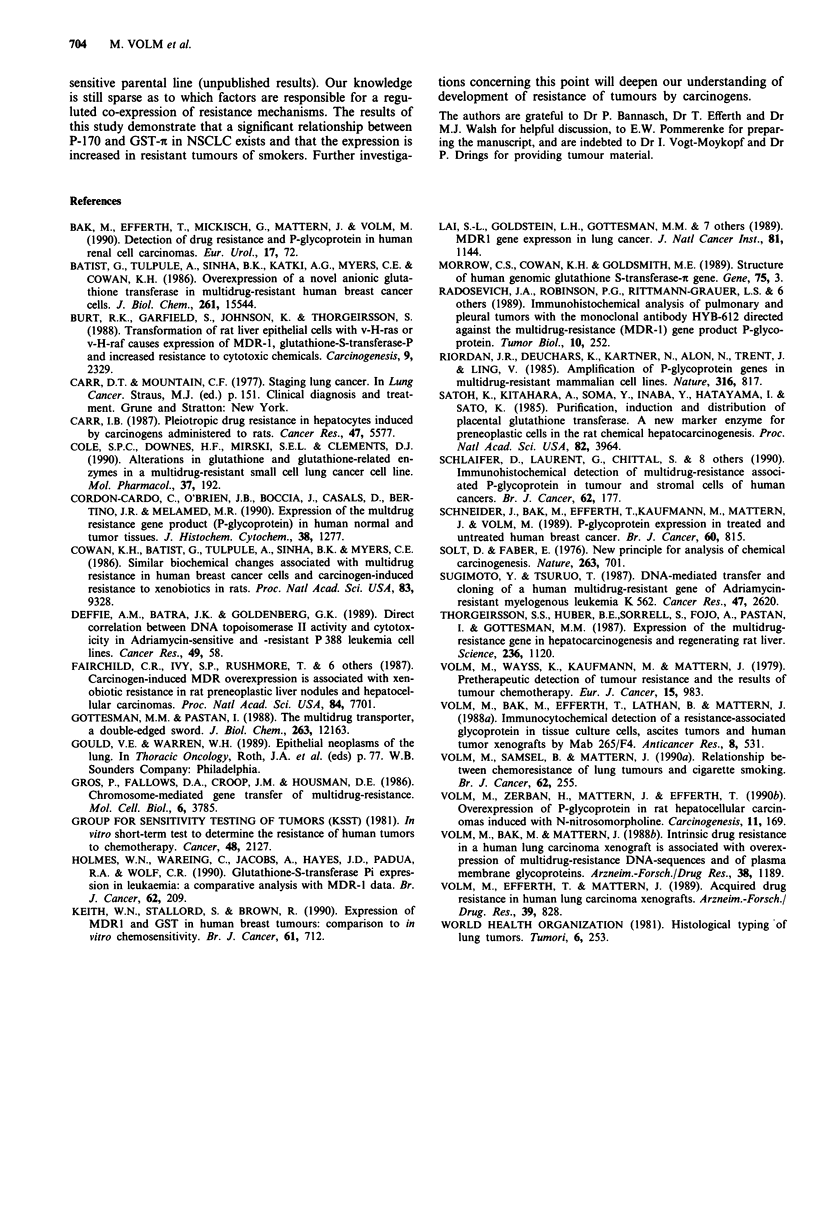# Overexpression of P-glycoprotein and glutathione S-transferase-pi in resistant non-small cell lung carcinomas of smokers.

**DOI:** 10.1038/bjc.1991.384

**Published:** 1991-10

**Authors:** M. Volm, J. Mattern, B. Samsel

**Affiliations:** German Cancer Research Center, Institute of Experimental Pathology, Heidelberg.

## Abstract

Ninety-four human non-small cell lung carcinomas (NSCLC) of previously untreated patients were analysed for the presence of P-glycoprotein (P-170) and glutathione S-transferase-pi (GST-pi) by means of immunohistochemistry. The expression of P-170 and GST-pi was compared with the results of doxorubicin resistance of the tumours in vitro and the smoking habits of the patients. A significant relationship between smoking habits of the patients and resistance of NSCLC was found (P = 0.007). Of the 72 tumours of smokers 57 (= 79%) were resistant, whereas of the 22 tumours of non-smokers only 11 (= 50%) showed resistance. Identical results were obtained when the analysis was restricted to patients with epidermoid lung carcinomas (P = 0.004). In contrast to these data, there exists no relationship between resistance and smoking for adenocarcinomas of the lung. Forty-two (= 58%) out of the 72 NSCLC of smokers expressed P-170, whereas out of 22 tumours of non-smokers only two tumours (= 9%) showed P-170 expression (P less than 0.0001). Similar results were obtained with epidermoid carcinomas (P = 0.004) and adenocarcinomas (P = 0.027). Fifty (= 69%) of 72 NSCLC of smokers revealed expression of GST-pi, whereas only nine (= 41%) of 22 tumours of non-smokers showed GST-pi expression (P = 0.015). Significant correlations also exist between resistance in vitro and expression of P-170 (P less than 0.0001) or expression of GST-pi (P less than 0.0001). Furthermore, a significant relationship between both proteins could be demonstrated (P less than 0.0001).


*Br. J. Cancer* (1991), **64**, 700–704 © Macmillan Press Ltd., 1991

### Overexpression of P-glycoprotein and glutathione S-transferase-π in resistant non-small cell lung carcinomas of smokers

M. Volm, J. Mattern & B. Samsel

*German Cancer Research Center, Institute of Experimental Pathology, Im Neuenheimer Feld 280, D-6900 Heidelberg, Germany.*

**Summary** Ninety-four human non-small cell lung carcinomas (NSCLC) of previously untreated patients were analysed for the presence of P-glycoprotein (P-170) and glutathione S-transferase-π (GST-π) by means of immunohistochemistry. The expression of P-170 and GST-π was compared with the results of doxorubicin resistance of the tumours *in vitro* and the smoking habits of the patients. A significant relationship between smoking habits of the patients and resistance of NSCLC was found ($P = 0.007$). Of the 72 tumours of smokers 57 ( = 79%) were resistant, whereas of the 22 tumours of non-smokers only 11 ( = 50%) showed resistance. Identical results were obtained when the analysis was restricted to patients with epidermoid lung carcinomas ($P = 0.004$). In contrast to these data, there exists no relationship between resistance and smoking for adenocarcinomas of the lung.

Forty-two ( = 58%) out of the 72 NSCLC of smokers expressed P-170, whereas out of 22 tumours of non-smokers only two tumours ( = 9%) showed P-170 expression ($P < 0.0001$). Similar results were obtained with epidermoid carcinomas ($P = 0.004$) and adenocarcinomas ($P = 0.027$). Fifty ( = 69%) of 72 NSCLC of smokers revealed expression of GST-π, whereas only nine ( = 41%) of 22 tumours of non-smokers showed GST-π expression ($P = 0.015$). Significant correlations also exist between resistance *in vitro* and expression of P-170 ($P < 0.0001$) or expression of GST-π ($P < 0.0001$). Furthermore, a significant relationship between both proteins could be demonstrated ($P < 0.0001$).

During the past few years the phenomenon of multidrug-resistance (MDR) has been thoroughly analysed and some of its molecular aspects clarified. The MDR-phenotype is characterised by cross-resistance between hydrophobic compounds without structural or functional similarities. The connection between MDR and the expression of a 170 kDa membrane glycoprotein (P-glycoprotein) has been clearly established in different tumour models (Riordan *et al.*, 1985). The most direct evidence of the role of the P-glycoprotein (P-170) in resistance has come from studies that have demonstrated that resistance can be conferred through transfer of genetic material encoding P-glycoprotein (Gros *et al.*, 1986; Sugimoto & Tsuruo, 1987). However, not all resistant tumours overexpress P-170 and refractoriness to chemotherapy can only partly be explained by P-170. Batist *et al.* (1986) described an elevated expression of glutathione S-transferase-π (GST-π ) in doxorubicin-resistant MCF-7 cells. Alterations in nuclear DNA topoisomerase II (Topo II) content have been also reported in doxorubicin-resistant P388 cells (Deffie *et al.*, 1989). Interestingly, Fairchild *et al.* (1987) observed an overexpression of both P-170 and GST-π in doxorubicin-resistant MCF-7 cells. This raises the question as to whether the expression of those two proteins may be under a common regulatory control.

In an earlier study with 160 human lung tumours (Volm *et al.*, 1990*a*) we demonstrated that there is a significant relationship between smoking and response to doxorubicin *in vitro*. Carcinomas of smokers tended to be resistant more frequently than carcinomas of non-smokers. Until now the mechanisms for the resistance of lung tumours are unknown. Therefore, we have analysed non-small cell lung carcinomas (NSCLC) of previously untreated patients for the presence of P-170 and GST-π by immunohistochemistry, a method which we have found useful for detection of P-170 in the past with different human tumours (Volm *et al.*, 1988*a*; Schneider *et al.*, 1989; Bak *et al.*, 1990). The current paper include 94 NSCLC of the earlier study (Volm *et al.*, 1990*a*) because alcohol-fixed samples used for immunostaining were only collected in the second part of the study.

Correspondence: M. Volm, Deutsches Krebsforschungszentrum, Institut für experimentelle Pathologie, Im Neuenheimer Feld 280, D-6900 Heidelberg, Germany.
Received 13 December 1990; and in revised form 28 May 1991.

### Materials and methods

### *Patients and tumours*

Patients with previously untreated NSCLC were surgically treated and fresh specimens of the tumours were fixed in alcohol and thereafter embedded in paraffin. The morphological classification of the bronchogenic carcinomas was based on the WHO study (1981) and comprised 48 epidermoid carcinomas, 34 adenocarcinomas and 12 large cell carcinomas. The histological classification of the tumours was carried out by two pathologists. The age distribution of the patients (81 men, 13 women) included one patient younger than 40 years, 11 patients between 40 and 49 years, 36 between 50 and 59, 35 between 60 and 69, and 11 older than 70 years (Table I). All patients were staged at time of surgery (pTNM). The classification of the stage was made according to the guidelines of the American Joint Committee for Cancer Staging and End Results Reporting (Carr & Mountain, 1977). Of the 94 patients 16 had stage I, ten stage II, and 68 had stage III tumours. Twenty-two of the patients were nonsmokers and 72 smokers. Nine of the smokers smoked one to ten cigarettes daily, twenty-four 11 to 20, twelve 21 to 30, thirteen 31 to 40, and five more than 40 cigarettes daily. The daily level of smoking could not be defined exactly for nine smokers.

### *Detection of resistance by the short term-test*

Most of the patients were treated by surgical procedures alone, or by combined surgical and radiation therapy. For this reason we used an *in vitro* test for determining the resistance of the tumours to drugs. The short-term test for predicting resistance to chemotherapy has been described previously (Volm *et al.*, 1979; Group for Sensitivity Testing of Tumours (KSST), 1981). Its basic feature is measurement of changes in the incorporation of radioactive nucleic acid precursors into tumour cells after addition of cytostatics. Briefly, the tumour cell suspensions are incubated with adriamycin (concentration $0.1-100\,\mu g\,ml^{-1}$) for 3 h at 37°C. Subsequently, $^3H$-uridine (concentration $2.5\,\mu C\,ml^{-1}$) is added during the last hour of incubation. Aliquots of the cell suspensions are pipetted onto filter discs, the acid-soluble radioactivity is extracted, and the incorporated activity measured by scintillation counting. Tumours were defined as being sensitive or resistant depending whether uridine uptake

**Table I** Patient characteristics ($n = 94$)

| *Clinical characteristics* | *No. of patients* |
|---|---|
| Age | |
| 40 | 1 |
| 40–49 | 11 |
| 50–59 | 36 |
| 60–69 | 35 |
| 70 | 11 |
| Sex | |
| male | 81 |
| female | 13 |
| Histology | |
| Epidermoid Ca | 48 |
| Adeno Ca | 34 |
| Large cell Ca | 12 |
| Stage | |
| I | 16 |
| II | 10 |
| III | 68 |
| Smoking habits | |
| Non-smokers | 22 |
| Smokers | 72 |

was inhibited by more or less than 35% respectively at a concentration of adriamycin of $10\,\mu g\,ml^{-1}$. This threshold was based on prior clinical correlations (Volm *et al.*, 1979; Group for Sensitivity Testing of Tumours, 1981).

### *Immunohistochemistry*

For detection of P-170 and GST-π the biotin-streptavidin-peroxidase method described previously was used (Volm *et al.*, 1988*a*). Briefly, alcohol-fixed, paraffin-embedded $5\,\mu m$ thick sections were deparaffinised. After preincubation with $H_2O_2$ (0.5%), unlabelled streptavidin, and non-immunised normal serum (dilution 1:10, 10 min, Dianova, Hamburg, Germany) the primary monoclonal antibodies were applied for 16 h at 4°C in a moist chamber. For detection of P-170 the monoclonal antibody JSB-1 ($10\,\mu g\,ml^{-1}$; Sanbio, Uden, Netherlands), for detection of glutathione S-transferase-π a rabbit antibody (dilution 1:2000; Satoh *et al.*, 1985; kindly provided by Dr P. Bannasch, German Cancer Research Center, Heidelberg) and for detection of Topo II anti-Topo II (1:500; kindly provided by Dr L. Liu, Baltimore, MD) were used. The antibody against GST-π was developed against the rat isoenzyme GST-P (Satoh *et al.*, 1985). Since rat GST-P and human GST-π isoenzymes share 85% amino acid sequence homology (Morrow *et al.*, 1989) we conclude that this antibody detects GST-π in human lung cancers. After three washing steps with PBS the cells were incubated with biotinylated sheep anti-mouse IgG (Amersham, Braunschweig, Germany) for detection of P-170 and donkey anti-rabbit IgG (Amersham) for detection of GST-π , and Topo II (30 min, dilution: 1:50 with 5% normal human serum). Afterwards, the streptavidin-biotinylated peroxidase complex (Amersham, 1:100, 30 min) was added. Peroxidase activity was visualised with 3-amino-9-ethylcarbazole (5–10 min) which gives a red-brown reaction product. Counterstaining was performed with haematoxylin and the sections were mounted with glycerol gelatin. Negative controls were done omitting the primary antibodies. Endogenous peroxidase activity of biopsy sections was quenched using 0.5% $H_2O_2$. Furthermore unspecific binding sites were blocked by incubation with normal sheep serum (P-170) and normal donkey serum (GST-π , Topo II), respectively (dilution 1:10, 10 min, Dianova). In addition, endogenous biotin may lead to potential problems in the application of streptavidin-based detection systems, therefore, unlabelled streptavidin (Amersham) was preincubated to suppress endogenous biotin-activity (dilution 1:50, 10 min). Because positive immunostaining was not only found in malignant cell populations but also in macrophages (Schlaifer *et al.*, 1990) we distinguished between tumour cells and macrophages using Mab CD68 (DAKO-CD68, KP1, Dakopatts, Copenhagen, Denmark). As positive controls, acetone- and alcohol-fixed multidrug-resistant CHO-cells and alcohol-fixed paraffin-embedded sections of P-170 and GST-π positive human kidney and colon carcinomas were used. In addition, we used a sensitive and resistant human myeloma cell line (8226/S; 8226/$DOX_{40}$) as negative and positive controls for P-170. Since 8226/S and 8226/$DOX_{40}$-cells have identical GST-π levels, we used 8226/$DOX_{40}$ as negative controls for GST-π .

Three observers (M.V., J.M., B.S.) independently evaluated and interpreted the results of immunohistochemical staining, without knowledge of the clinical data of each patient and the results of the resistance test. The immunohistochemical staining was expressed according to a semiquantitative scale which we have previously established with a series of multidrug-resistant cell lines. The tumour samples were graded as zero when there was complete absence of plasma membrane staining or cytoplasmatic staining. The immuno-positive tumour samples were graded as one-plus and two-plus according to the degree of immunohistochemical staining of cells in all areas of the specimens examined. The highest degree of positivity found in any area of tissue section was recorded.

**Table II** Epidermoid carcinoma of the lung

| *No.* | *Stage* | *Smoking habits* | *In vitro test* | *Staining P-170* | *GST* |
|---|---|---|---|---|---|
| 1 | II | NS[a] | sens[c] | O | O |
| 2 | I | NS | sens | O | O |
| 3 | III | NS | sens | O | O |
| 4 | III | NS | sens | O | O |
| 5 | III | NS | sens | O | + + |
| 6 | III | NS | res[d] | O | + |
| 7 | I | NS | res | O | + |
| 8 | III | NS | res | O | + + |
| 9 | III | NS | res | O | + + |
| 10 | I | NS | res | + | + + |
| 11 | III | S[b] | sens | O | O |
| 12 | I | S | sens | O | O |
| 13 | III | S | sens | O | + + |
| 14 | III | S | sens | + | O |
| 15 | III | S | res | O | O |
| 16 | II | S | res | O | O |
| 17 | II | S | res | O | O |
| 18 | I | S | res | O | O |
| 19 | I | S | res | O | O |
| 20 | III | S | res | O | O |
| 21 | III | S | res | O | O |
| 22 | III | S | res | O | + |
| 23 | III | S | res | O | + |
| 24 | II | S | res | O | + + |
| 25 | III | S | res | O | + + |
| 26 | III | S | res | O | + + |
| 27 | III | S | res | + | + |
| 28 | III | S | res | + | + |
| 29 | III | S | res | + | + |
| 30 | III | S | res | + | + |
| 31 | II | S | res | + | + + |
| 32 | III | S | res | + | + + |
| 33 | III | S | res | + | + + |
| 34 | III | S | res | + | + + |
| 35 | II | S | res | + | + + |
| 36 | III | S | res | + | + + |
| 37 | III | S | res | + | + + |
| 38 | III | S | res | + | + + |
| 39 | I | S | res | + | + + |
| 40 | III | S | res | + | + + |
| 41 | III | S | res | + | + + |
| 42 | III | S | res | + + | O |
| 43 | III | S | res | + + | + + |
| 44 | III | S | res | + + | + + |
| 45 | III | S | res | + + | + + |
| 46 | III | S | res | + + | + + |
| 47 | I | S | res | + + | + + |
| 48 | III | S | res | + + | + + |

[a]NS = non-smoker; [b]S = smoker; [c]sens = sensitive; [d]res = resistant; O = no staining; + = weakly positive, + + = strongly positive.

### Results

In the present study, 94 human non-small cell lung carcinomas (NSCLC) of previously untreated patients were analysed for the presence of P-glycoprotein (P-170) and glutathione S-transferase-π (GST-π ) by means of immunohistochemistry. Clinical data of the patients and characteristics of the tumours are given in Table I. In Table II to Table IV the immunohistochemical data are listed together with the results of resistance of the tumours *in vitro* and the smoking habits of the patients. Of the 48 patients with epidermoid lung carcinomas, 38 were smokers and 10 non-smokers (Table II). Of the 48 tumours, 39 ( = 81%) were resistant and nine ( = 19%) were sensitive to doxorubicin in the test. Twenty-four tumours ( = 50%) showed positivity for P-170 and 33 ( = 69%) for GST-π .

**Table III** Adenocarcinoma of the lung

| *No.* | *Stage* | *Smoking habits* | *In vitro test* | *Staining P-170* | *GST* |
|---|---|---|---|---|---|
| 1 | II | NS[a] | sens[c] | O | O |
| 2 | III | NS | sens | O | O |
| 3 | III | NS | sens | O | O |
| 4 | III | NS | sens | O | O |
| 5 | III | NS | sens | O | O |
| 6 | III | NS | sens | O | O |
| 7 | I | NS | res[d] | O | O |
| 8 | III | NS | res | O | O |
| 9 | III | NS | res | O | O |
| 10 | III | NS | res | O | Q |
| 11 | III | NS | res | O | + + |
| 12 | III | NS | res | + + | + + |
| 13 | I | S[b] | sens | O | O |
| 14 | III | S | sens | O | O |
| 15 | III | S | sens | O | O |
| 16 | III | S | sens | O | O |
| 17 | III | S | sens | O | O |
| 18 | I | S | sens | O | + |
| 19 | III | S | sens | O | + |
| 20 | III | S | sens | O | + + |
| 21 | III | S | sens | + | + |
| 22 | III | S | sens | + | + |
| 23 | II | S | res | O | O |
| 24 | III | S | res | O | O |
| 25 | III | S | res | O | O |
| 26 | III | S | res | O | O |
| 27 | III | S | res | + | + |
| 28 | III | S | res | + | + + |
| 29 | III | S | res | + + | + |
| 30 | III | S | res | + + | + + |
| 31 | I | S | res | + + | + + |
| 32 | I | S | res | + + | + + |
| 33 | III | S | res | + + | + + |
| 34 | III | S | res | + + | + + |

[a]NS = non-smoker; [b]S = smoker; [c]sens = sensitive; [d]res = resistant; O = no staining; + = weakly positive, + + = strongly positive.

**Table IV** Large cell carcinoma of the lung

| *No.* | *Stage* | *Smoking habits* | *In vitro test* | *Staining P-170* | *GST* |
|---|---|---|---|---|---|
| 1 | III | S[a] | sens[b] | O | + + |
| 2 | III | S | res[c] | O | O |
| 3 | III | S | res | O | + + |
| 4 | I | S | res | + | O |
| 5 | II | S | res | + | + |
| 6 | I | S | res | + | + |
| 7 | I | S | res | + | + + |
| 8 | III | S | res | + | + + |
| 9 | III | S | res | + | + + |
| 10 | III | S | res | + | + + |
| 11 | II | S | res | + + | + + |
| 12 | III | S | res | + + | + + |

[a]S = smoker; [b]sens = sensitive; [c]res = resistent; O = no staining; + = weakly positive; + + = strongly positive.

Of the 34 patients with adenocarcinomas, 22 were smokers and 12 were non-smokers (Table III). Eighteen out of 34 tumours showed resistance *in vitro* ( = 59%) whereas 16 tumours ( = 41%) were sensitive. Eleven tumours ( = 32%) were positive for P-170 and 16 ( = 47%) for GST-π . All 12 patients with large cell carcinomas of the lung were smokers (Table IV). All tumours except one showed resistance *in vitro*. Nine tumours ( = 75%) showed expression of P-170 and ten ( = 83%) expression of GST-π .

In order to examine whether a relationship exists between smoking habits of patients, resistance of tumours *in vitro*, and expression of P-170 or GST-π , we analysed the data by Fisher exact test. We found a significant relationship between smoking habits of the patients with NSCLC and resistance *in vitro* ($P = 0.007$) (Table V). Carcinomas of smokers were more frequently resistant than carcinomas of non-smokers. Of the 72 non-small cell lung carcinomas of smokers, 57 ( = 79%) were resistant, whereas of the 22 tumours of non-smokers only 11 ( = 50%) showed resistance. This relationship is more apparent for the epidermoid lung carcinomas. Of the 38 tumours of smokers, 34 ( = 89%) were resistant and of the ten tumours of non-smokers only five tumours ( = 50%) were resistant ($P = 0.004$). In contrast to these data, there exists no relationship between resistance and smoking for adenocarcinomas of the lung. This may be expected because adenocarcinomas are said to be less frequently associated with smoking than are epidermoid lung carcinomas.

The relationship between expression of P-170 and GST-π , respectively and smoking habits of the patients, is also shown in Table V. Forty-two ( = 58%) out of 72 non-small cell lung carcinomas of smokers showed expression of P-170, whereas out of 22 tumours of non-smokers only two ( = 9%) revealed P-170 expression. This relationship is significant ($P < 0.0001$). A similar significant relationship was found for epidermoid carcinomas ($P = 0.004$) and adenocarcinomas ($P = 0.027$). Fifty NSCLC ( = 69%) of smokers out of 72 tumours revealed strong expression of GST-π. In contrast, only nine ( = 41%) out of 22 NSCLC of non-smokers showed GST-π expression. The relationship between smoking habits of the patients and overexpression of GST-π is significant ($P = 0.015$). Similar results are obtained when the analysis is restricted to just those patients with epidermoid lung carcinomas and adenocarcinomas of the lung, but the results are not significant (Table V). We found significant correlations between resistance measured *in vitro* and expression of P-170 ($P < 0.0001$) or expression of GST-($P < 0.0001$) (Table VI).

Moreover, we correlated the expression of P-170 and GST-π and also found a significant relationship ($P < 0.0001$) (Table VII). A significant inter-relationship between P-170 or GST-π

**Table V** Relationship between resistance, expression of P-glycoprotein and glutathione S-transferase and smoking habits of patients with lung carcinomas

| *Tumours* | | *Non-smokers* | *Smokers* | *P-value** |
|---|---|---|---|---|
| All tumours | Sensitive | 11 | 15 | $P = 0.007$ |
| | Resistant | 11 | 57 | |
| | P-170 neg. | 20 | 30 | $P < 0.0001$ |
| | P-170 pos. | 2 | 42 | |
| | GST neg. | 13 | 22 | $P = 0.015$ |
| | GST pos. | 9 | 50 | |
| Epidermoid lung carcinoma | Sensitive | 5 | 4 | $P = 0.004$ |
| | Resistant | 5 | 34 | |
| | P-170 neg. | 9 | 15 | $P = 0.004$ |
| | P-170 pos. | 1 | 23 | |
| | GST neg. | 4 | 11 | n.s. |
| | GST pos. | 6 | 27 | |
| Adenocarcinoma | Sensitive | 6 | 11 | n.s. |
| | Resistant | 6 | 11 | |
| | P-170 neg. | 11 | 12 | $P = 0.027$ |
| | P-170 pos. | 1 | 10 | |
| | GST neg. | 9 | 9 | n.s. |
| | GST pos. | 3 | 13 | |

*Fisher exact test.

**Table VI** Relationship between resistance and P-glycoprotein or glutathione S-transferase expression of non-small cell lung carcinomas

| | *Sensitive* | *Resistant* | *P-value** |
|---|---|---|---|
| P-170 neg. | 23 | 27 | $P<0.0001$ |
| P-170 pos. | 3 | 41 | |
| GST neg. | 18 | 17 | $P<0.0001$ |
| GST pos. | 8 | 51 | |

*Fisher exact test.

**Table VII** Relationship between P-glycoprotein expression and glutathione S-transferase expression

| | *P-170 neg.* | *P-170 pos.* | *P-value** |
|---|---|---|---|
| GST negative | 32 | 3 | $P<0.0001$ |
| GST positive | 18 | 41 | |

*Fisher exact test.

and age of the patients, the number of cigarettes smoked, the stage or size of the tumours was not found.

The expression of topoisomerase II was also assessed immunohistochemically. However, we did not find an association to either drug resistance of tumours or smoking habits of patients (data not shown).

### Discussion

The fact that exposure to chemical carcinogens results in resistant cells is well known and has been shown again recently during the past few years (Carr, 1987). In the present study, we demonstrate that NSCLC of smokers are more frequently resistant than tumours of non-smokers. Seventy-nine per cent of the tumours of smokers were resistant, whereas only 50% of the tumours of non-smokers revealed resistance. These results are significant ($P = 0.007$). Identical results were obtained when the analysis was restricted to patients with epidermoid lung carcinomas. In contrast, we found no relationship between doxorubicin-resistance *in vitro* and smoking for adenocarcinomas of the lung. This may be expected because adenocarcinomas are less frequently associated with smoking than are epidermoid lung carcinomas (Gould & Warren, 1989).

Until now, the mechanisms for the resistance of lung tumours were unknown but might be multifactorial. Interestingly, there exists a remarkable parallel between the biochemical changes with carcinogen resistance and multidrug-resistance. Thorgeirsson *et al.* (1987) and Fairchild *et al.* (1987) reported that multidrug-resistance gene transcripts are elevated in preneoplastic and neoplastic nodules in the rat liver during carcinogenesis induced according to the protocol of Solt-Faber (Solt & Faber, 1976). We also used a model of chemical carcinogenesis in which only one carcinogen, N-nitrosomorpholine, was administered and investigated whether overexpression of P-glycoprotein also takes place in hepatocellular carcinomas (Volm *et al.*, 1990*b*). As shown by our experiments, the overexpression of the multidrug-resistance gene is apparent in hepatocellular carcinomas even after withdrawal of the carcinogen. It seems that the multidrug-resistance gene belongs to a programmed set of detoxifying mechanisms that is also expressed during carcinogenesis and might be important for lung tumours caused by smoking.

In the past few years several mechanisms for the development of multidrug resistant cell lines *in vitro* have been identified, for instance overexpression of P-glycoprotein (P-170), glutathione S-transferase-π (GST-π) and reduction of DNA topoisomerase II (Topo II). P-170 appears to function as a transporter which extrudes a broad spectrum of compounds from the cell (Gottesman & Pastan, 1988). Glutathione S-transferases represent a family of isoenzymes which conjugate glutathione with various xenobiotics and may play a role in detoxification. Topo II induces double strand breaks during the DNA replication and downregulation of Topo II activity circumvents cytotoxic effects. There are some hints that these detoxifying systems may share common regulatory elements. In the present study we investigated the overexpression of P-170, GST-π and Topo II in NSCLC and its relationship to doxorubicin-resistance *in vitro* and smoking habits of the patients. We found a significant relationship between the overexpression of P-170 or GST-π , and resistance of the tumours or smoking habits of the patients ($P<0.0001$). The overexpression of these proteins are more frequent in resistant NSCLC and in tumours of smokers. In contrast, we did not find an association of Topo II to either drug resistance of tumours or smoking habits of the patients. Thus, Topo II expression is not linked with resistance of NSCLC of smokers.

In the present study, 44 out of 94 non-small cell lung carcinomas ( = 47%) revealed an expression of P-170. Interestingly, many tumours show a weak immunostaining for P-170. We could not find differences between low levels and higher levels of P-170 with regard to our resistance-results *in vitro*. This indicates that low levels of P-170 are sufficient to produce a resistance phenotype. This also demonstrates the importance of very sensitive detection systems e.g. immunohistochemistry, for assessing P-170 expression in human tumours. Radosevich *et al.* (1989) found P-170-expressing cells in 100 out of 131 non-small cell lung carcinomas ( = 76%) by immunohistochemical techniques. Lai *et al.* (1989) demonstrated a weak expression of P-glycoprotein mRNA in 14 out of 24 lung tumours ( = 58%). In contrast to these results, other authors have only occasionally detected expression of the MDR gene in lung tumours (Cordon-Cardo *et al.*, 1990). We recently investigated the intrinsic and acquired resistance of human epidermoid lung cancer xenografts grown in nude mice and found a correlation between expression of P-170 and degree of resistance (Volm *et al.*, 1988*b*, 1989). As shown by Lai *et al.* (1989) expression of MDR1 RNA of lung cancers was not significantly different from that of corresponding normal lung tissue samples. In own analyses we also found no differential expression of P-170 and MDR1 RNA between normal lung tissue and lung cancers in a limited number of probes (data not shown).

In the present study we correlated the expression of P-170 and GST-π and found a significant correlation ($P<0.0001$). Cowan *et al.* (1986) reported that P-170 and GST-π are both overexpressed in doxorubicin-resistant MCF-7 cells. Burt *et al.* (1988) demonstrated that transformation of rat liver cells with v-H-*ras* or v-*raf* oncogenes results in a MDR-phenotype and an elevated expression of the MDR-1 and the GST-π gene. Keith *et al.* (1990) found a weak correlation between expression of MDR1 and GST-π in human breast cancer samples suggesting that common mechanisms may be involved. To determine if there is an association between GST-π and MDR1 expression in human leukaemia, Holmes *et al.* (1990) have investigated their expression in haemopoietic cells of untreated and treated patients. They found no significant correlation in patients with myelodysplastic syndrome or in patients with acute myeloblastic leukaemia, but a positive association between GST-π and MDR1 expression in patients with chronic lymphatic leukaemia. In own experiments we have observed a correlation between GST-π and MDR1 expression in samples of human kidney carcinoma but not in human leukaemia or in breast cancer (Efferth, T., Mattern, J., Volm, M; in preparation). This indicates that the co-regulation of resistance mechanisms is not a common feature and might be dependent on the tumour type. In fact, other authors did not find a co-expression of P-170 and GST-π. Cole *et al.* (1990) described a doxorubicin-resistant small-cell lung cancer cell line (H 69 AR) with elevated activities of GST-π but without overexpression of P-170. In earlier investigations we have analysed doxorubicin- and daunomycin-resistant sublines of murine Sa 180 and L 1210 which were grown *in vivo* as ascites. In contrast to the resistant Sa 180 tumour lines which show an overexpression of P-170 and GST and a reduction of Topo II, the resistant L 1210 lines shown only an overexpression of P-170 and the levels of GST and Topo II are unchanged compared with the sensitive parental line (unpublished results). Our knowledge is still sparse as to which factors are responsible for a regulated co-expression of resistance mechanisms. The results of this study demonstrate that a significant relationship between P-170 and GST-π in NSCLC exists and that the expression is increased in resistant tumours of smokers. Further investigations concerning this point will deepen our understanding of development of resistance of tumours by carcinogens.

The authors are grateful to Dr P. Bannasch, Dr T. Efferth and Dr M.J. Walsh for helpful discussion, to E.W. Pommerenke for preparing the manuscript, and are indebted to Dr I. Vogt-Moykopf and Dr P. Drings for providing tumour material.

### References

BAK, M., EFFERTH, T., MICKISCH, G., MATTERN, J. & VOLM, M. (1990). Detection of drug resistance and P-glycoprotein in human renal cell carcinomas. *Eur. Urol.*, **17**, 72.

BATIST, G., TULPULE, A., SINHA, B.K., KATKI, A.G., MYERS, C.E. & COWAN, K.H. (1986). Overexpression of a novel anionic glutathione transferase in multidrug-resistant human breast cancer cells. *J. Biol. Chem.*, **261**, 15544.

BURT, R.K., GARFIELD, S., JOHNSON, K. & THORGEIRSSON, S. (1988). Transformation of rat liver epithelial cells with v-H-ras or v-H-raf causes expression of MDR-1, glutathione-S-transferase-P and increased resistance to cytotoxic chemicals. *Carcinogenesis*, **9**, 2329.

CARR, D.T. & MOUNTAIN, C.F. (1977). Staging lung cancer. In *Lung Cancer*. Straus, M.J. (ed.) p. 151. Clinical diagnosis and treatment. Grune and Stratton: New York.

CARR, I.B. (1987). Pleiotropic drug resistance in hepatocytes induced by carcinogens administered to rats. *Cancer Res.*, **47**, 5577.

COLE, S.P.C., DOWNES, H.F., MIRSKI, S.E.L. & CLEMENTS, D.J. (1990). Alterations in glutathione and glutathione-related enzymes in a multidrug-resistant small cell lung cancer cell line. *Mol. Pharmacol.*, **37**, 192.

CORDON-CARDO, C., O'BRIEN, J.B., BOCCIA, J., CASALS, D., BERTINO, J.R. & MELAMED, M.R. (1990). Expression of the multdrug resistance gene product (P-glycoprotein) in human normal and tumor tissues. *J. Histochem. Cytochem.*, **38**, 1277.

COWAN, K.H., BATIST, G., TULPULE, A., SINHA, B.K. & MYERS, C.E. (1986). Similar biochemical changes associated with multidrug resistance in human breast cancer cells and carcinogen-induced resistance to xenobiotics in rats. *Proc. Natl Acad. Sci. USA*, **83**, 9328.

DEFFIE, A.M., BATRA, J.K. & GOLDENBERG, G.K. (1989). Direct correlation between DNA topoisomerase II activity and cytotoxicity in Adriamycin-sensitive and -resistant P 388 leukemia cell lines. *Cancer Res.*, **49**, 58.

FAIRCHILD, C.R., IVY, S.P., RUSHMORE, T. & 6 others (1987). Carcinogen-induced MDR overexpression is associated with xenobiotic resistance in rat preneoplastic liver nodules and hepatocellular carcinomas. *Proc. Natl Acad. Sci. USA*, **84**, 7701.

GOTTESMAN, M.M. & PASTAN, I. (1988). The multidrug transporter, a double-edged sword. *J. Biol. Chem.*, **263**, 12163.

GOULD, V.E. & WARREN, W.H. (1989). Epithelial neoplasms of the lung. In *Thoracic Oncology*, Roth, J.A. *et al.* (eds) p. 77. W.B. Sounders Company: Philadelphia.

GROS, P., FALLOWS, D.A., CROOP, J.M. & HOUSMAN, D.E. (1986). Chromosome-mediated gene transfer of multidrug-resistance. *Mol. Cell. Biol.*, **6**, 3785.

GROUP FOR SENSITIVITY TESTING OF TUMORS (KSST) (1981). *In vitro* short-term test to determine the resistance of human tumors to chemotherapy. *Cancer*, **48**, 2127.

HOLMES, W.N., WAREING, C., JACOBS, A., HAYES, J.D., PADUA, R.A. & WOLF, C.R. (1990). Glutathione-S-transferase Pi expression in leukaemia: a comparative analysis with MDR-1 data. *Br. J. Cancer*, **62**, 209.

KEITH, W.N., STALLORD, S. & BROWN, R. (1990). Expression of MDR1 and GST in human breast tumours: comparison to *in vitro* chemosensitivity. *Br. J. Cancer*, **61**, 712.

LAI, S.-L., GOLDSTEIN, L.H., GOTTESMAN, M.M. & 7 others (1989). MDR1 gene expresson in lung cancer. *J. Natl Cancer Inst.*, **81**, 1144.

MORROW, C.S., COWAN, K.H. & GOLDSMITH, M.E. (1989). Structure of human genomic glutathione S-transferase-π gene. *Gene*, **75**, 3.

RADOSEVICH, J.A., ROBINSON, P.G., RITTMANN-GRAUER, L.S. & 6 others (1989). Immunohistochemical analysis of pulmonary and pleural tumors with the monoclonal antibody HYB-612 directed against the multidrug-resistance (MDR-1) gene product P-glycoprotein. *Tumor Biol.*, **10**, 252.

RIORDAN, J.R., DEUCHARS, K., KARTNER, N., ALON, N., TRENT, J. & LING, V. (1985). Amplification of P-glycoprotein genes in multidrug-resistant mammalian cell lines. *Nature*, **316**, 817.

SATOH, K., KITAHARA, A., SOMA, Y., INABA, Y., HATAYAMA, I. & SATO, K. (1985). Purification, induction and distribution of placental glutathione transferase. A new marker enzyme for preneoplastic cells in the rat chemical hepatocarcinogenesis. *Proc. Natl Acad. Sci. USA*, **82**, 3964.

SCHLAIFER, D., LAURENT, G., CHITTAL, S. & 8 others (1990). Immunohistochemical detection of multidrug-resistance associated P-glycoprotein in tumour and stromal cells of human cancers. *Br. J. Cancer*, **62**, 177.

SCHNEIDER, J., BAK, M., EFFERTH, T.,KAUFMANN, M., MATTERN, J. & VOLM, M. (1989). P-glycoprotein expression in treated and untreated human breast cancer. *Br. J. Cancer*, **60**, 815.

SOLT, D. & FABER, E. (1976). New principle for analysis of chemical carcinogenesis. *Nature*, **263**, 701.

SUGIMOTO, Y. & TSURUO, T. (1987). DNA-mediated transfer and cloning of a human multidrug-resistant gene of Adriamycin-resistant myelogenous leukemia K 562. *Cancer Res.*, **47**, 2620.

THORGEIRSSON, S.S., HUBER, B.E.,SORRELL, S., FOJO, A., PASTAN, I. & GOTTESMAN, M.M. (1987). Expression of the multidrug-resistance gene in hepatocarcinogenesis and regenerating rat liver. *Science*, **236**, 1120.

VOLM, M., WAYSS, K., KAUFMANN, M. & MATTERN, J. (1979). Pretherapeutic detection of tumour resistance and the results of tumour chemotherapy. *Eur. J. Cancer*, **15**, 983.

VOLM, M., BAK, M., EFFERTH, T., LATHAN, B. & MATTERN, J. (1988*a*). Immunocytochemical detection of a resistance-associated glycoprotein in tissue culture cells, ascites tumors and human tumor xenografts by Mab 265/F4. *Anticancer Res.*, **8**, 531.

VOLM, M., SAMSEL, B. & MATTERN, J. (1990*a*). Relationship between chemoresistance of lung tumours and cigarette smoking. *Br. J. Cancer*, **62**, 255.

VOLM, M., ZERBAN, H., MATTERN, J. & EFFERTH, T. (1990*b*). Overexpression of P-glycoprotein in rat hepatocellular carcinomas induced with N-nitrosomorpholine. *Carcinogenesis*, **11**, 169.

VOLM, M., BAK, M. & MATTERN, J. (1988*b*). Intrinsic drug resistance in a human lung carcinoma xenograft is associated with overexpression of multidrug-resistance DNA-sequences and of plasma membrane glycoproteins. *Arzneim.-Forsch./Drug Res.*, **38**, 1189.

VOLM, M., EFFERTH, T. & MATTERN, J. (1989). Acquired drug resistance in human lung carcinoma xenografts. *Arzneim.-Forsch./Drug. Res.*, **39**, 828.

WORLD HEALTH ORGANIZATION (1981). Histological typing of lung tumors. *Tumori*, **6**, 253.